# Crystallization of Poly(ethylene terephthalate): A Review

**DOI:** 10.3390/polym16141975

**Published:** 2024-07-10

**Authors:** Maria Laura Di Lorenzo

**Affiliations:** National Research Council, Institute for Polymers, Composites and Biomaterials, CNR-IPCB, Via Campi Flegrei, 34, 80078 Pozzuoli, NA, Italy; marialaura.dilorenzo@cnr.it

**Keywords:** poly(ethylene terephthalate), polyester, crystallization, kinetics, structure

## Abstract

Poly(ethylene terephthalate) (PET) is a thermoplastic polyester with excellent thermal and mechanical properties, widely used in a variety of industrial fields. It is a semicrystalline polymer, and most of the industrial success of PET derives from its easily tunable crystallization kinetics, which allow users to produce the polymer with a high crystal fraction for applications that demand high thermomechanical resistance and barrier properties, or a fully amorphous polymer when high transparency of the product is needed. The main properties of the polymer are presented and discussed in this contribution, together with the literature data on the crystal structure and morphology of PET. This is followed by an in-depth analysis of its crystallization kinetics, including both primary crystal nucleation and crystal growth, as well as secondary crystallization. The effect of molar mass, catalyst residues, chain composition, and thermo-mechanical treatments on the crystallization kinetics, structure, and morphology of PET are also reviewed in this contribution.

## 1. Introduction

Poly(ethylene terephthalate) (PET) is one of the most widely used polymers, as it ranks fourth in the worldwide production of plastics [1,2] and is the polymer most used to produce food containers (especially bottles for water and soft drinks) and textile fibers [3,4]. PET is a semicrystalline polyester, with a glass transition temperature (*T*_g_) of 69 °C [5,6] and an equilibrium melting point $T_{m}^{{}^{\circ}}$ = 280 °C [6,7]. It has slow crystallization kinetics, which implies high energy consumption and long time required to attain a semicrystalline product, but also allows for the easy production of an amorphous, transparent polymer [8].

**Figure 1:**
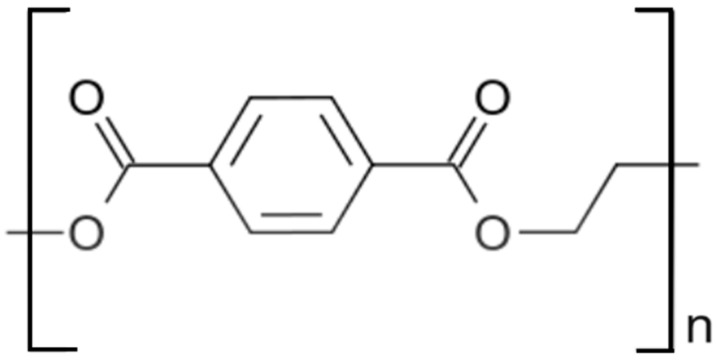
Repeating unit of PET.

The repeating unit of PET is illustrated in Figure 1. This polymer was first synthesized in 1946 and commercially introduced in 1953, initially as textile fiber, and then for the production of films and blow-molded bottles [9]. The wide application range of PET derives from its excellent chemical stability, low weight, thermal resistance, and mechanical properties when semicrystalline, coupled to its relatively low cost and easy recyclability [2,9,10].

Currently, PET is industrially produced via the initial synthesis of a prepolymer, bis(2-hydroxyethyl) terephthalate (BHET), followed by polycondensation. BHET prepolymer can be produced via the esterification of terephthalic acid (TPA) and ethylene glycol (EG) or via the transesterification of dimethyl terephthalate and EG. [11]. The reaction route that uses terephthalic acid is generally preferred, because of its faster reaction kinetics and the lower cost of the monomer, compared to dimethyl terephthalate [12]. Depending on the extent of the polycondensation reaction, polymers with various molar masses can be attained and used for specific applications. PET with a molar mass of 15,000–25,000 is used in textile applications, whereas a molar mass higher than 30,000 is needed for injection or blow molding applications [13]. All monomers for PET synthesis are generally produced from petroleum-based resources; however, fully bio-based PET, attained from annually renewable feedstocks, is also commercially available [14].

PET is often viewed as an environmentally friendly polyester, despite being non-degradable in normal conditions. This is due to the possibility of producing the polymer from bio-based monomers, mentioned above, and to the economically feasible technologies that have been developed to recover/recycle post-consumer PET as an alternative to its traditional disposal into landfills. These processes comprise primary (re-extrusion), secondary (mechanical), tertiary (chemical), and quaternary (energy recovery) recycling [15,16,17,18,19,20].

Primary and secondary recycling imply the re-extrusion of production scraps or re-extrusion after sorting and separation from collected waste, respectively [15]. The re-extrusion and, in general, melt reprocessing of PET leads to chain scission with a reduction in molecular weight, which negatively impacts the mechanical properties, melt viscosity and impact resistance of the polymer [20]. In chemical recycling (a tertiary process), PET is depolymerized to its original components, and then repolymerized [19]. Various technologies have been developed for the depolymerization of PET, with methanolysis and glycolysis mostly applied in large-scale industrial facilities [15]. The quaternary process is energy recovery, which consists of the incineration of post-consumer PET in a furnace. This process is generally preferred in cases of highly contaminated PET, when other methods of separation and recovery are not suitable [15]. Energy produced from incineration can be converted into electricity and the residuals from the incinerator, free from toxicity hazards, can be safely disposed in landfills [18].

With its high melting temperature (ca. 250 °C), semicrystalline PET retains its desirable mechanical properties when exposed to elevated temperatures up to 175 °C, i.e., close to the onset of crystal melting [21]. A great variety of microstructures can be developed in PET by changing its crystallization conditions. Crystal size and shape, volume fraction, orientation, and perfection can be varied through control of the crystallization process. The study of the crystallization process and microstructure development is intriguing and provides deeper insight into the effect of process parameters on the structure, development, and, ultimately, the properties of the PET end product.

## 2. Crystal Structure

**Figure 2:**
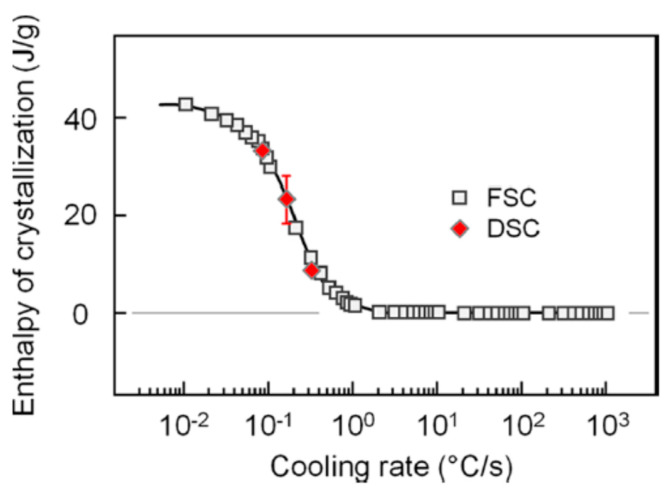
Enthalpy of the crystallization of PET as a function of the cooling rate, as measured by FSC (squares) and DSC (red diamond symbols). Reprinted with permission from Ref. [25]. Copyright (2015) American Chemical Society.

PET is a semicrystalline polyester with a rather slow crystallization rate. In fact, it can be easily attained in an amorphous form by quenching from its molten state [22,23,24,25], since a cooling rate of 1 K/s is sufficient to prevent crystal formation in PET [25]. This is exampled in Figure 2, which reports the enthalpy of the crystallization of a PET with an intrinsic viscosity of 0.74 dL/g as a function of its cooling rate, measured by a combination of conventional (DSC) and fast scanning chip (FSC) calorimetry: cooling at rates of 1 K/s or higher leads to no measurable enthalpy, which indicates that no crystallization takes place upon cooling at these rates. PET crystallizes only in part when cooled at rates between 1 and 10^−2^ K/s, whereas completion of the phase transition is attained by cooling the polymer at rates of 10^−2^ K/s or lower [25].

**Figure 3:**
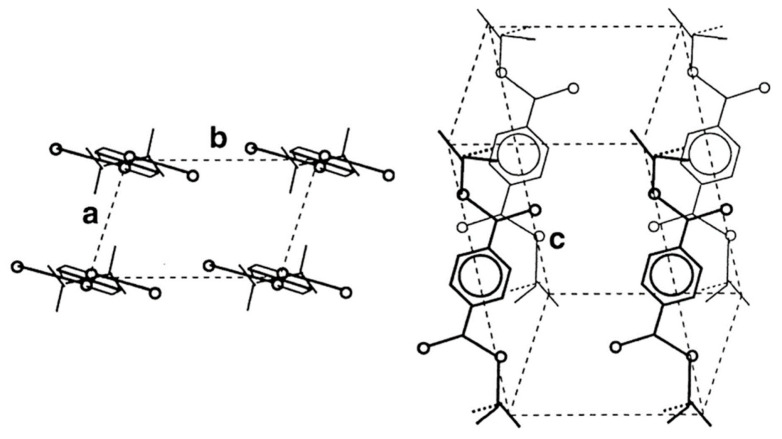
Scheme of a triclinic unit cell of PET, with *a* = 4.56 Å, *b* = 5.94 Å, *c* = 10.75 Å, α = 98.5°, β = 118°, γ = 112°. Adapted from Ref. [26], Copyright (2000), with permission from Elsevier.

Upon crystallization, PET chains organize within a triclinic cell containing one repeating unit, as sketched in Figure 3 [26]. The triclinic arrangement has *a* = 4.56 Å, *b* = 5.94 Å, *c* = 10.75 Å, α = 98.5°, β = 118°, and γ = 112°; this results in a crystal density of 1.455 g/cm^3^, sizably higher than that of the amorphous polymer, which has a density of 1.335 g/cm^3^ [27]. Upon stretching, this crystal structure can be deformed, causing changes in the *d*-spacings of the (100) and (010) planes [28], and, in most drawn PET fibers, the crystals have their *c*-axes not parallel to the fiber axes, but tilted [28,29].

**Figure 4:**
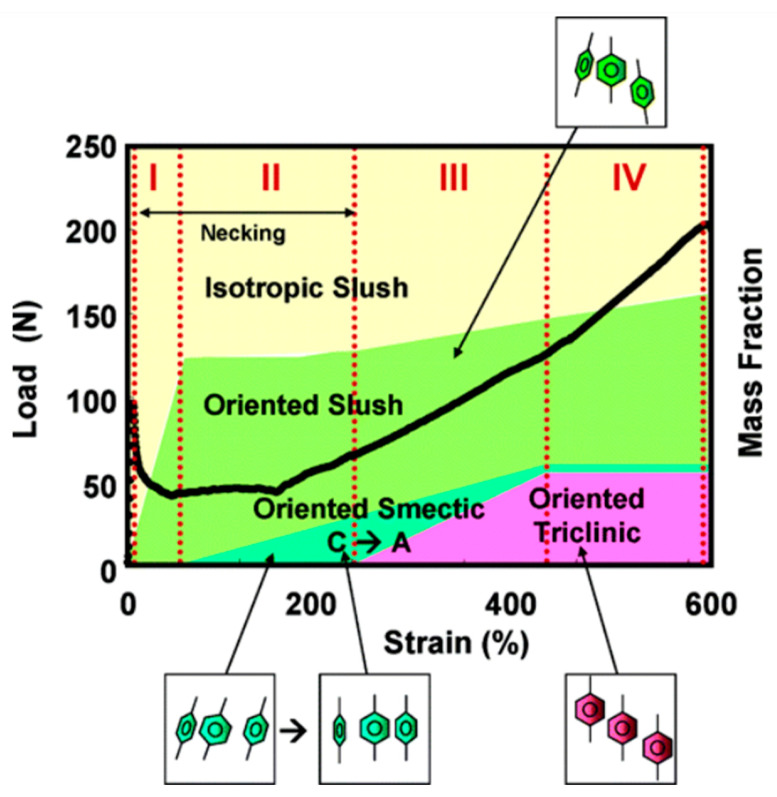
Evolution of PET structure upon drawing, with changes sketched as four steps (I to IV). Reprinted with permission from Ref. [46]. Copyright (2005) American Chemical Society.

Compared to other polyesters [30], PET displays limited crystal polymorphism. Besides the triclinic crystals, only mesophase arrangements have been reported. These mesophases can be attained by drawing an initially amorphous polymer at temperatures below the glass transition temperature (*T*_g_), as first highlighted by Bonart [31,32,33,34,35,36,37]. This mesophase was initially identified as having a smectic A structure (chain axis parallel to the stretching direction without a tilting arrangement) [38], with later investigations revealing more detailed information on the chain organization, structure, and properties of PET mesophases [39,40,41,42,43,44,45]. Wide-angle and small-angle X-ray scattering analyses upon tensile deformation below *T*_g_ (70 °C) of an initially amorphous PET disclosed the sequence of its strain-induced phase transformations/transitions: when stretched, the initially amorphous polymer initially develops isotropic slush (i.e., a mixture of amorphous and nematic phases), and then oriented slush, which then progressively transforms to smectic C, quasi-smectic A, and finally to triclinic crystal [46,47,48]. This structure development pathway is illustrated in Figure 4: the yield point is related to the onset of the isotropic slush to oriented slush transformation, the “plastic deformation” zone is linked to the transformation of the oriented slush to a smectic phase, with the onset of the stress increase that is related to the smectic C to quasi-smectic A transformation, and the strain-hardening process is dominated by the smectic–triclinic crystalline transition [46].

When heated, the smectic array of PET chains transforms to a crystalline triclinic structure above 80 °C [38]. This transition was first probed by small- and wide-angle X-ray diffraction analyses of cold-drawn PET films annealed at various temperatures, from 50 to 240 °C, for times ranging from 10 to 10^4^ s. The appearance of a diffuse SAXS reflection at 70 °C before the development of the triclinic crystal suggested the existence of a precursor state, attained by a molecular tilting in the smectic phase with minimum cooperative displacement [38]. Further heating of the triclinic structure did not lead to the appearance of a different polymorph until melting [49].

## 3. Crystallization Kinetics

The crystallization of PET generally proceeds via a three-stage process, as is typical in polymer crystallization: it starts with primary nucleation, which is followed by crystal growth and secondary crystallization/perfection [50]. In primary nucleation, crystal nuclei develop. This can occur via the statistical fluctuation of chain segments in the melt (homogeneous nucleation) or can be eased by heterogeneities dispersed in the polymer (heterogeneous nucleation). The latter mechanism generally prevails upon melt processing, where heterogeneous nucleation starts on the surfaces, cavities, or cracks of impurities. Crystalline lamellae then grow on these nuclei to develop three-dimensional superstructures, most commonly spherulites, as often reported for PET [51,52,53]. Secondary crystallization and crystal perfection may occur after spherulite impingement, but also during their growth [54,55,56,57]. During secondary crystallization, crystal growth takes place in geometrically restricted areas, which produces topological constraints and, consequently, a higher percentage of segments immobilized in proximity of the crystal surfaces. The additional constraints in the amorphous chain segments coupled to the crystals may lead to the vitrification of a rigid amorphous fraction (RAF), as detailed in a separate paragraph below [58,59].

### 3.1. Homogeneous and Heterogeneous Nucleation in PET

Small aggregates of few parallel chain segments that reach a critical size and become sufficiently stable can act as homogeneous crystal nuclei able to initiate crystal growth on their surface [55,56]. The critical size of homogeneous nuclei depends on the crystallization temperature (*T*_c_), and more specifically on the difference between the equilibrium melting point ($T_{m}^{{}^{\circ}}$) and the crystallization temperature, a parameter named supercooling ($∆T=T_{m}^{{}^{\circ}}-T_{c}$). An increase in supercooling, i.e., a decrease in *T*_c_, implies a smaller size of the critical nuclei able to start crystallization, but, at the same time, the reduced temperature leads to kinetic restraints caused by the increased energy needed for the transport of chain segments to the growth front or a decrease in the length scale of mobility [50,55,56,60,61,62].

A quantitative analysis of homogeneous crystal nucleation is not straightforward, because crystal nuclei are of nanometer size and their formation involves a too little energy to be directly measurable; therefore, indirect methods are generally used. The most common methods rely on an analysis of the kinetics of crystal growth after nuclei formation or on an estimation of the density of the developed crystals or of the measurement of the melting of the crystals grown from the stabilized nuclei [63]. Tammann’s two-stage crystal nuclei development method is often applied to attain information on the kinetics of the formation of homogeneous crystal nuclei in polymers. The first stage consists of the development of crystal nuclei in the temperature range where homogeneous nucleation prevails, and the second stage involves an estimation of their growth to crystals as an indirect quantification of the number of developed nuclei. This procedure, initially proposed for the analysis of nucleation in organic liquids and inorganic glasses [64,65], has also been used for the quantitative analysis of the kinetics of homogeneous nucleation for a wide variety of polymers [50,66,67,68,69,70,71,72], including PET [25,73,74].

The two-stage method was initially used to prove the increased nucleation density in an initially amorphous PET which was annealed at temperatures around *T*_g_ (60–80 °C, the nuclei development stage) for 15 h and then isothermally crystallized at 115 °C for 30 min (crystal growth stage) [73]. The samples annealed around *T*_g_ displayed a much higher number of spherulites compared to the polymer directly heated to the crystallization temperature, which provided proof of the development of additional crystal nuclei during the annealing step that preceded the isothermal crystallization at 115 °C [73].

**Figure 5:**
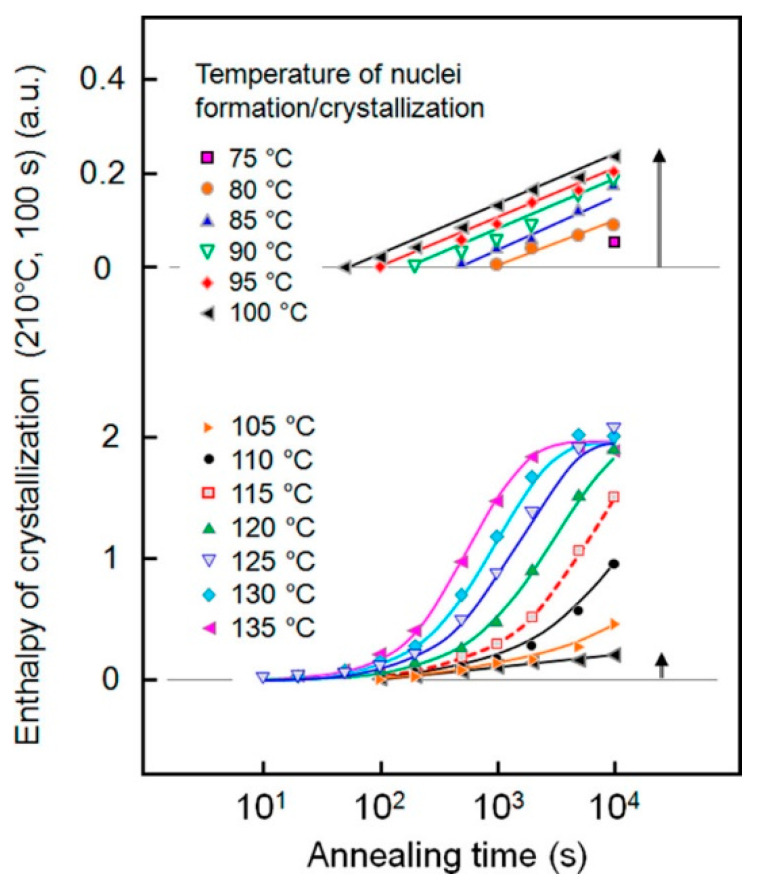
Enthalpy of cold crystallization at 210 °C for 100 s as a function of the annealing time at the nucleation-stage temperatures indicated in the legend. Data attained via nucleation at 100 °C (black triangles) are repeated in both parts of the figure. Reprinted with permission from Ref. [25]. Copyright (2015) American Chemical Society.

Tammann’s two-stage approach was exploited to determine the rate of homogeneous nuclei development in PET using fast scanning chip calorimetry (FSC). The formation of nuclei at low temperatures was monitored via the measurement of the subsequent cold crystallization at higher temperatures, together with an analysis of the stability of the homogeneous nuclei upon temperature increases. Quantitative data are illustrated in Figure 5, which presents the enthalpy of the cold crystallization of PET at 210 °C for 100 s as a function of the time of annealing at temperatures ranging from 75 to 135 °C [25]. An increase in the temperature results in a faster rate of nuclei formation, with a varied trend above 100 °C, where measurable crystal growth overlaps with nuclei formation and stabilization. Homogeneous nuclei partially melt during the transfer from the temperature of their formation to the growth temperature, and their incomplete dissolution allows for the development of slightly larger and more stable nuclei. This way, the initially formed nuclei keep their overcritical size up to 100 °C, or even higher above the nucleation temperature, by the continuous increase in their size [25].

The analysis of crystal nucleation kinetics is of importance not only to tailor the production of semicrystalline PET, but also to favor the attainment of an amorphous polymer, as the low crystallization rate of PET may be advantageous for fabricating transparent products. In fact, the possibility of easily fabricating amorphous, transparent PET products favored the commercial exploitation of this polymer, which is now largely used in injection stretch–blow molding for the production of bottles for water or carbonated soft drinks. In fact, polymer crystallization upon blow molding leads to opaque bottles not suitable for the market of bottled water or soft drinks [75,76]. The easy of control of PET crystallization is also of importance in the textile industry, because a lower crystallinity improves the productivity and dyeability of PET fibers [77], as well as in the production of foams [78]: PET can crystallize when exposed to supercritical CO_2_, the most commonly used blowing agent [79], but CO_2_ can be dissolved only in the amorphous areas of the polymer. In a semicrystalline polymer, the fraction of the sample that can undergo bubble formation is reduced by its decreased CO_2_ content, while crystallization also results in increased matrix stiffness, which also limits foam expansion [80,81]. However, the slow crystallization rate of PET can also complicate industrial processing when a semicrystalline product is desired. For instance, injection-molded PET articles often have poor mechanical properties, unless long molding cycles, not applicable in industrial contexts, are used [82,83]. To improve crystallization kinetics, PET grades are often formulated with nucleating agents (heterogeneous nucleation) and/or plasticizers. Nucleating agents promote the onset of crystal growth, whereas plasticizers reduce *T*_g_, favoring the transport of chain segments towards the growing crystals, at equal crystallization temperatures [53].

The most common nucleating agents for PET include inorganic, organic, organic–inorganic hybrid molecules, and macromolecules. Some inorganic nucleating agents commonly used for other polymers, like silica [84,85,86], clay [87,88,89,90], calcium carbonate [91], titanium dioxide [92], mica [93], and micro- or nano-glass flakes [94], do not display good compatibility with PET [95]. However, poor matrix–filler compatibility can be improved, e.g., by the surface coating of the filler, thus turning the additives into efficient nucleating agents [96]. This is the case of calcium carbonate nanoparticles coated with stearic acid, where the coating provided improved the compatibility of the nanoparticles and, in turn, their efficacy as nucleating agents for PET [96]. On the other hand, some organic molecules used as heterogeneous nucleants, like sodium salts of carboxylic acids, can induce the degradation of the PET chain during processing due to attack of the ester by the sodium carboxylate [97,98,99], a process also named “chemical nucleation” [99]. Organic–inorganic hybrids used as nucleating agents for PET mostly include polyhedral oligomeric silsesquioxane (POSS) [100], modified graphene oxide [101,102], and organic silica quantum dots [103], and all have been shown to display high compatibility with PET [104]. A few polymers have also been reported as efficient heterogeneous nucleating agents for PET, like polyammide 6,6 [105], liquid crystal copolyester [106], and ionomers [107].

### 3.2. Crystal Growth

PET crystallizes from the melt with a spherulitic morphology [108,109,110], and has a maximum crystallization rate around 160–180 °C [111,112]. The crystallization kinetics of PET display several peculiarities: besides the effects commonly seen in polymer crystallization, like thermal history, crystallization temperature, and the presence of additives like nucleating agents or plasticizers [55], the crystallization rate of PET is also largely affected by a number of specific features, mainly including its molar mass, the presence of branches due to side reactions occurring during polymerization, and the catalyst system used during polycondensation [112].

For a thorough analysis of the crystallization rate of PET, the thermal treatment needed to erase its previous thermal history must be carefully selected. In polymer crystallization analysis, this is usually achieved by heating a sample above the melting point for a time sufficient to remove all crystals or crystal-like aggregates that may remain in the melt and induce accelerated crystallization due to self-seeding nucleation [113]. In PET, exposure to high temperatures, even when lower than the melting point, may lead to chain degradation, which can affect subsequent crystallization kinetics. Initial studies on this process showed that a prolonged annealing of PET at 250 °C (i.e., below its equilibrium melting point) may result in transesterification reactions that involve the molecular chain segments in amorphous regions. These rearrangements lead to the formation of tie molecules and a loss of chain mobility, as well as to an increase in the overall molar mass [114]. The kinetics of transesterification in PET, investigated by small-angle neutron scattering, revealed that ester interchange reactions are fast in the melt and occur at a slower rate at 15 °C below the melting point [115,116]. Transesterification was suggested to decrease the number of chain entanglements and promote crystallization in PET [117] and was also linked to the peculiar ability of PET to develop high-density crystals upon prolonged annealing above melting (several months at 290 °C) [116].

**Figure 6:**
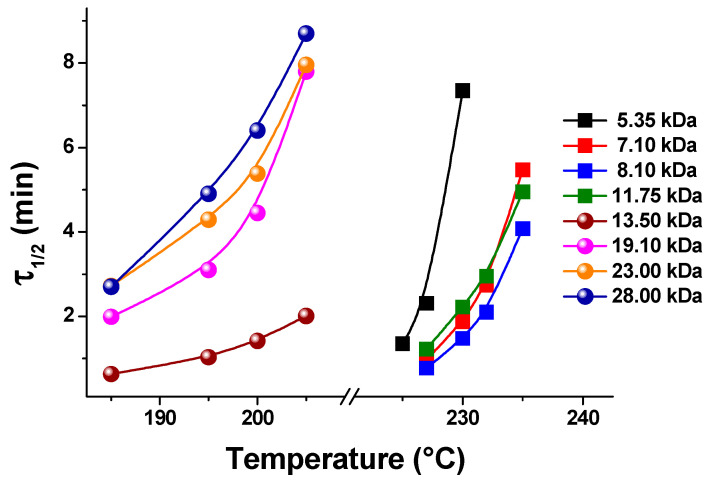
Half-time of crystallization (τ_½_) of PET grades with different molar masses [120,121].

Molar mass is known to largely influence the crystallization rate of polymers, as it results in a shift in their glass transition and equilibrium melting temperatures that in turn vary the crystallization window [55,118,119]; moreover, a variation in molar mass also affects the motion of chain segments toward the crystal growth front, which can become more difficult with the increase in chain length, due to entanglements [55,119]. PET presents an additional, peculiar dependence on molar mass [109,110,111,112,113,114,115,116,117,118,119,120,121,122,123,124,125], as shown in Figure 6, where literature data [120,121] on the half-time of crystallization (τ_½_), i.e., the time needed to reach half of the final crystallinity, are plotted as function of the crystallization temperature (*T*_c_): for molar mass above 8–9 kDa, τ_½_ values indicate that the overall crystallization rate decreases with increasing chain length, whereas, for low molar mass polymers, the phase transition becomes slower for PET grades with shorter chains. This results in an atypical maximum in the crystallization rate noted for PET with a molar mass around 8–9 kDa. This maximum arises from a balance between two opposite effects: (i) the decrease in free energy for the formation of critical nuclei for subsequent crystal growth with the increase in molar mass and (ii) the increase in the energy needed for chain transport due to the higher viscosity. The difference in the activation energies of the smallest and largest molar mass chains is ascribed to their different crystallization mechanisms, which vary with molar mass: short chains assume a stretched conformation upon crystallization (high activation energy), whereas the high molar mass polymers crystallize with a folded chain conformation, which has a low activation energy and results in a faster phase transition rate [120]. Such mechanisms have also been reported for a few other polymers, like polyethylene [119], poly(ethylene oxide) [122], and poly(ε-caprolactone) [123].

**Figure 7:**
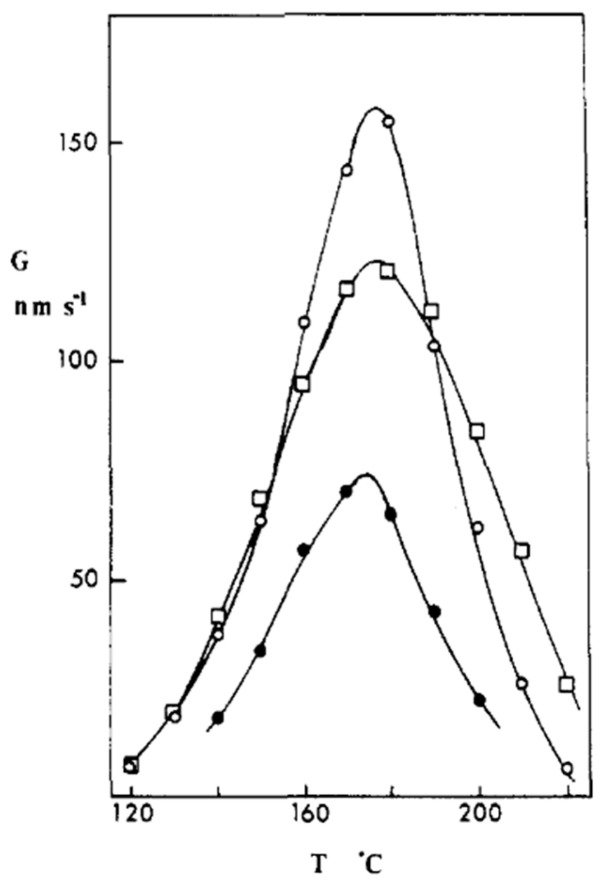
Spherulite growth rates of different PET grades. Reprinted with permission from Ref. [117]. Copyright (1989) American Chemical Society.

The temperature dependence of the rate of the spherulite growth (*G*) of PET is presented in Figure 7, which collects data reported in the literature by a few authors [124]. All sets of data display a maximum in crystal growth rate at 176–177 °C, and the sizable variation in crystallization rates arises from the differences in the chain features of the various grades, which often do not allow researchers to define a clear trend with specific parameters, e.g., molar mass, as often reported for other semicrystalline polymers [55]. This is due to some peculiarities of PET crystallization, whose kinetics can be markedly affected by a number of additional chain features, and most importantly by differences in diethylene glycol content [125], as discussed in detail in the following paragraph.

## 4. Influence of Chain Features

The synthesis of PET from dimethyl terephthalate and ethylene glycol leads to the formation of diethylene glycol (DEG, 2,2-oxydiethanol) as a side product. DEG has a high boiling point (245 °C), higher than that of ethylene glycol (198 °C), and the similar reactivity of the two molecules results in the partial formation of copolymers containing a small amount of diethylene glycol terephthalate (DEGT). For this reason, PET commercial grades are indeed copolymers that generally comprise around 2–5 mol%. of DEG [125,126,127]. The DEG comonomer largely affects the thermal properties of PET: besides its effects on light, hydrolytic, thermal, and oxidative degradation, and the improved dyeing ability of the fibers [125,128,129,130,131], higher amounts of DEG result in a decrease in the glass transition and melting temperature of 1–3 K for every mol% of DEG [126,129], and this leads to a varied crystallization window and kinetics [125,126,127,132,133].

The influence of DEG on the crystallization rate of PET is strongly influenced by the effective supercooling ($∆T=T_{m}^{{}^{\circ}}-T_{c}$). At low temperatures (cold crystallization, high supercooling), discordant results have been reported: some authors claimed an increase in crystallization rate with DEG content [134], whereas no sizable effect was noted by other authors [127,135]. At low temperatures, molecular motion is the rate-determining step in polymer crystallization, hence the higher flexibility in the aliphatic chain portions containing DEG favors phase transition [54,134]. This is counterbalanced by the enhanced irregularity of the chain with increasing DEG content, which hinders phase transition [126,127,135]. Conversely, upon melt crystallization (high temperatures, low supercooling), the influence of DEG is more evident, because the effect of a decreased equilibrium melting point, and hence of the degree of supercooling, is more pronounced [127]. This results in a sizably lower phase transition rate [126,127,134], as is typical for multicomponent polymer systems [136].

Crystallinity generally decreases with DEG content due to the segregation of DEG-containing chain portions to the amorphous region. This causes larger intralamellar amorphous areas with a lower density than that of the crystalline parts [125,137]. Copolymerization with DEG has no measurable effect on unit cell dimensions, which indicates that DEG units are excluded from the crystallites, or that their inclusion is limited to lattice defects [125,131,137].

PET-based copolymers containing other acyclic glycol co-units have also been synthesized, with the goal of tailoring the crystallization kinetics. Examples of acyclic glycol co-units which were found to decrease the crystallization and crystallinity of PET include 1,3-propanediol [138,139], 2,2-dimethyl-1,3-propanediol [139], 2,2-diethyl-1,3-propanediol [139], 2,2-butyl-ethyl-1,3-propanediol, [139,140], 1,4-butanediol [141], (2S,3S)-2,3-dimethoxy-1,4-butanediol [142], 1,4-cyclohexylene dimethylol [143,144,145,146], and *cis*/*trans* 2,2,4,4-tetramethyl-1,3-cyclobutanediol [147].

Other co-units have been introduced into PET chains to tailor their crystallization kinetics. Copolymerization with isophthalic acid (IPA) reduces the melting point, crystallinity, and crystallization rate of PET [77]. Its quiescent melt crystallization and strain-induced crystallization rates are markedly decreased upon copolymerization with IPA [148,149,150]. The incorporation of 15 mol% IPA comonomer leads to a melting point decrease of 20 K [151], and no crystallization is observed with an IPA fraction of more than 20% [152]. Substituted isophthalic comonomers were also tested, like dimethyl 5-nitroisophthalate [153,154], 5-tert-butylisophthalic acid [155,156,157,158,159], 5-adamantylisophthalic acid [159], and sodium 5-sulfoisophthalate [160]. Overall, the size of the attached group on the aromatic ring has a major influence on the thermal properties of the copolymer [161], and in some cases some enhancement of the crystallization rate was reported. This is the case for 5-tert-butylisophthalic comonomer [157,158], which can fasten the overall crystallization of PET when present in small amounts (up to 5%), possibly by facilitating nucleation [161].

Copolymerization with 2,5-furandicarboxylic acid (FDCA), which has a similar aromatic structure to terephthalic acid and is also bio-based, has also been envisaged as an efficient route to tailor the thermal properties of PET: both crystallization rate and crystallinity decrease with the comonomer content, and the decrease is more marked upon melt than cold crystallization [162,163]. At equal comonomer contents, copolymerization with FDCA leads a higher reduction in its crystallization rate than IPA, thanks to the higher rigidity and polarity of FCDA, which hinders chain packing during crystallization. Moreover, copolymerization with FDCA results in copolyesters with a higher *T*_g_, higher tensile modulus, and better optical clarity and gas barrier properties, compared to those attained with IPA [164].

Other comonomers have also been tested to tailor the crystallization kinetics of PET. They include, among others, phtalic acid [165], isosorbide (1,4:3,6-dianhydrohexitols) [166], and sulfonates [167,168], all leading to a decreased crystallization rate of PET.

Residual catalysts can also affect the crystallization rate of PET [9,169], with some quantitative data also reported in the literature [170,171,172,173,174,175,176]. Several types of catalysts can be used to synthesize PET, mostly organometallic compounds based on antimony, germanium, and titanium, with antimony trioxide being the most used one [177]. The type of catalyst can largely influence the crystallization of PET. Being difficult to remove from the polymer, the residual catalyst particles may act as heterogeneous nuclei, facilitating the onset of crystal growth [178]. This holds especially for insoluble catalysts [172,176], since soluble catalysts have no measurable role as heterogeneous nuclei [9].

However, the possible role as a nucleating agent is not the only effect of a residual catalyst on the crystallization rate of PET. The type of catalysis determines the synthesis reaction route, leading to copolymerization with DEG (whose influence on phase transition kinetics is discussed above), the formation of branches or other side reactions, and terminal groups [9,177]. This overlaps with other structural features that also affect the crystallization rate of PET, e.g., molar mass, which results in the often contradicting results reported in the literature. For instance, an antimony-based catalyst is reported to enhance the phase transition rate in Ref. [169], whereas in Ref. [157] the same catalyst is claimed to have appreciable effects on the crystallization kinetics of PET only when present in amounts much higher than those commonly used.

Similar to other polyesters [179], chain branching in PET may have a complex influence on crystallization behavior. Besides the branches resulting from side reactions during polycondensation, branched PET can be purposely produced to improve processing, foaming, mechanical resistance, or to facilitate recycling. Therefore, these processes, commonly named chain extensions, are widely studied in the literature, with various technologies developed to date, like reactor polycondensation (RP), solid-state polycondensation (SSP), and reactive extrusion (REX) [180,181,182,183]. Most importantly, in the context of this review, chain branching largely affects the thermal properties of PET and, in turn, its crystallization behavior. Long-chain branching may have only a limited influence on *T*_g_, whereas short-chain branches often result in a lower *T*_g_ due to the increase in free volume [180]. Regarding the melting point, the literature data point to a decrease in *T*_m_, coupled with lower crystallinity, with increasing branching content [180,184].

To understand the influence of chain branching on the crystallization of PET, multiple effects need to be taken into account. The most relevant ones refer to the varied chain mobility induced by branches, the effect of branching on melting point and glass transition, chain entanglements, and branches with varied chemistry (e.g., larger DEG contents) [185]. Each branching site represents a point of irregularity in the macromolecular chain which should be excluded from the crystals. While this hinders crystallization, the presence of small concentrations of branches, kinks, and linear disruptions may also enhance PET crystallization by favoring nucleation [186,187]. Similarly, a decrease in crystallinity due to chain extension, linked to entanglements that complicate chain folding, has been reported [188], as well as the opposite effect at high temperatures [189]. Therefore, both faster and slower phase transition rates ascribed to side chains have been reported in the literature for PET [185,186,187,188,189,190,191,192,193], possibly due to the wide variety of chain topologies that can be produced, which adds to the multiple effects of other chain features, like comonomer content, catalyst residue, etc., largely complicating an unequivocal assessment of each parameter.

## 5. Crystallization of PET in Blends and Composites

Besides copolymerization with different co-units, or the incorporation of reactive functional groups for chain branching, as detailed in the previous paragraph, blending with other high molar mass polymers and the addition of fillers are also methods commonly used to modify the crystallization behavior of PET [194]. In polymer blends, crystallization and, in general, thermal, mechanical, and barrier properties, are determined by the miscibility of the components [136], or, in cases of non-miscible blends, can be improved by the addition of a compatibilizer [195]. In the specific case of a polymer with reactive functional groups in the main chain, like PET, its crystallization and thermal properties can also be altered by ester-interchange reactions that, depending on the extent of reaction, may turn an immiscible and non-compatible binary blend into a compatible system or even to a miscible one [161].

A typical example is blends of PET with poly(ethylene 2,6-naphthalate) (PEN): the two polymers are immiscible; however, in cases of the reactive blending of the two homopolymers, PET/PEN copolymers develop, which can enhance the compatibility of the blend components [196]. With a low extent of transesterification, PET and PEN independently crystallize [197], as often observed in immiscible polymer pairs [136], whereas, above a critical degree of transesterification, a marked influence of each component was reported, with crystallization largely affected by the blend composition and strain-induced crystallization that result in the co-crystallization of the two polymers [198]. For a transesterified PET/PEN (50/50) blend, the extent of the reaction leads to a transition from a completely amorphous, incompatible, but well-dispersed PET/PEN blend to a miscible and crystallizable block copolymer, and then to a non-crystallizable copolymer with an essentially random microstructure [199].

Similar results were reported for the reactive blending of an equimolar immiscible blend of PET with bisphenol-A polycarbonate (PC) [200]. The progress of their transreactions initially led to their conversion to crystallizable block copolymers and then to a random, amorphous PET/PC copolymer. The annealing of the amorphous copolymer below the melting point of PET allowed for the restoration of their crystallization ability via sequential reordering [200], with the non-crystallizable comonomer units coupled to the crystalline domains that were converted to crystallizable units through interchange reactions [201,202,203].

Similar results were reported for blends of PET with other polymers, where the influence of reactive blending and the extent of the interchain reactions play a major role in crystallization behavior, as claimed, among others, for blends with poly(lactic acid) [204,205], poly(butylene terephthalate) [206,207], polyamides [208,209], or poly(ethylene vinyl acetate) [210].

Efforts have also been made to regulate the crystallization rate of PET through the inclusion of fillers in PET-based composites. These fillers generally act as heterogeneous nucleating agents, favoring the onset of crystal growth and a reduced crystal size, thus enhancing not only the overall crystallization rate, but also material’s properties [211,212]. The main parameters that determine the efficacy of a filler in enhancing the crystallization rate of PET are linked to the homogeneity of its dispersion and adhesion/interaction with the PET matrix [213]. This is crucial especially for nanosized fillers, due to the easy of clustering of the nanoparticles. In such cases, nanoparticle coatings or compatibilizers need to be used to avoid nanoparticle aggregation and enhance dispersion and adhesion with the polymer and, in turn, crystallization kinetics [96].

PET composites containing a variety of fillers able to promote crystal nucleation have been developed. The most commonly used fibers able to enhance the crystallization rate of PET include glass fibers [214], carbon fibers [215], and natural fibers [216,217]. As for nanocomposites, several types of nanoparticles have been reported to enhance the crystallization kinetics and crystallinity of PET, like clays [86,218], SiO_2_ [103,219], modified graphene oxide [220], graphene quantum dots [221], multiwalled carbon nanotubes [222,223,224,225], polyhedral oligomeric silsesquioxanes [100,226,227], calcium carbonate [96], silicon carbide [228], and metal–organic frameworks [95,229].

## 6. Influence of Liquid and Gases

Plasticizers, which may be contained in commercial PET formulations [63,230,231,232,233], can also influence crystallization kinetics, as typically reported for semicrystalline polymers [55]. Plasticizers are low molar mass molecules able to reduce viscosity, *T*_g_, and the elastic modulus, thus imparting higher flexibility and improving processability [234,235]. Plasticizers may have multiple effects on crystallization: a decrease in *T*_g_ shifts the crystallization window to lower temperatures, while increased chain mobility facilitates the transport of chain segments to the growing crystals, especially at low temperatures, but also reduces the equilibrium melting point, thus leading to varied supercooling at parity of crystallization temperature. In other words, a plasticizer can facilitate crystallization via reducing the energy terms related to chain mobility, and also the nucleation and growth rate, thanks to its lower supercooling at an equivalent temperature [236].

Due to the high reactivity of the functional groups in PET, functional plasticizers may react with the polymer and form copolymers. The influence of copolymerization on plasticizer efficacy, including the effect on crystallization kinetics, was specifically investigated for poly(ethylene glycol) (PEG) oligomers of a molar mass of 1 kDa via solid-state polymerization (SSP) of PET with either PEG, or end-capped PEG [237]. While plain PEG formed copolymers with PET, its end-capped analogue was merely dispersed in the amorphous phase, with no reaction detected upon SSP. However, both compounds resulted in an similar reduction in *T*_g_ and in the crystallization behavior of PET [237]. Other plasticizers were found to be effective in enhancing the crystallization kinetics of PET, like dioctyl phthalate [238] or tall oil fatty acid [239].

PET can absorb moisture from the environment, and water molecules can promote PET degradation at high temperatures; hence, the polymer is commonly dried before melt processing [240]. On the other hand, water also plasticizes PET, aiding polymer processing at low temperatures [241,242,243,244]. Water molecules reduce the *T*_g_ of PET, leading to a decrease of 15 °C in the fully saturated polymer compared to the dry one [241,243]. Water can increase the cold crystallization rate of PET, favoring the onset of crystal growth [243,245], with larger lamellar thickness and crystal size in the wet polymer compared to the dry material [246]. Conversely, upon stretching around *T*_g_, water suppresses crystallization, despite promoting chain mobility [244,247]. The strain-induced crystallization of PET around *T*_g_ proceeds via the prior formation of a mesophase, which acts as a structural intermediate to promote nucleation. When PET is saturated with water, an intermediate nanophase arrangement is not observed during the whole stretch process. Moreover, in dried PET, no strain softening was reported before mesophase ordering, whereas, in wet PET, yield and strain softening were shown to occur before the onset of crystallization, with the respective stress and modulus that decrease with water content [244]. This was interpreted as a higher water content corresponding to lower stress, higher chain mobility, and lower crystallinities [244].

**Figure 8:**
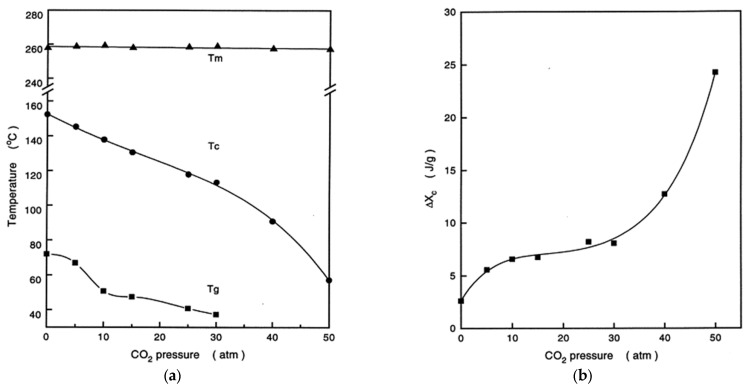
Influence of CO_2_ pressure on (**a**) glass transition (*T*_g_), cold crystallization (*T*_c_), and melting temperatures (*T*_m_) of PET; (**b**) crystal fraction (Δ*X*_c_) of PET. Reprinted from Ref. [250]. Copyright (1999), with permission from Elsevier.

Another low molar mass molecule widely studied for its influence on the crystallization kinetics of PET is carbon dioxide [248,249,250,251,252,253,254,255]. The huge amount of research conducted in this context derives from the frequent use of CO_2_ as foaming agent, being CO_2_ the most widely used foaming agent for polyesters [254,255,256,257,258,259,260]. CO_2_ can plasticize PET, with sizable effects on *T*_g_ and crystallization kinetics. The influence of CO_2_ on glass transition, cold crystallization, and melting temperatures of PET is quantified on the left side of Figure 8, with the right side of the same figure presenting the variation of PET crystallinity as a function of CO_2_ pressure [260]. The sorption of CO_2_ has marked effects on *T*_g_ and cold crystallization, and a minor influence on melting point, which indicates that the same *T*_c_ corresponds to a similar supercooling. Moreover, an increase in CO_2_ pressure induces a higher dissolution of the gas into the polyester, which has a very marked effect on crystallization due to the significantly enhanced chain mobility that favors the transport of PET chains to the crystals [260].

## 7. Coupling between Crystalline and Amorphous Parts: The Rigid Amorphous Fraction

Polymer crystallization is often accompanied by a partial vitrification of amorphous chain segments [58]. Semicrystalline polymers have a three-phase structure, with one crystal fraction and two amorphous fractions. Besides the mobile amorphous fraction (MAF) made of the chain portions that mobilize at *T*_g_, there is another amorphous nanophase, named the rigid amorphous fraction (RAF), that is made of the amorphous chain portions connected to the crystal lamellae. The constraints exerted by the crystals lead to a reduced mobility of the coupled, nearby amorphous chain portions that constitute the rigid amorphous fraction. This results in a reduced mobility of the RAF compared to the MAF, with the RAF devitrifying at temperatures higher than the *T*_g_ of the MAF [59].

The RAF is established at the basal planes of crystals [261]. The amount of RAF (*w*_RAF_) is generally quantified by calorimetry, via the measurement of the mobile amorphous fraction (*w*_MAF_) at the completion of *T*_g_ and of the crystallinity (*w*_C_), as *w*_RAF_ = 1 − *w*_C_ − *w*_MAF_ [58,59].

The RAF plays a crucial role in determining the properties of semicrystalline polymers [59]. The RAF affects the barrier properties of PET, with quantitative data on its permeability to oxygen available in the literature, including the solubility coefficients of O_2_ in PET, which are higher in the MAF than in the RAF [262]. The RAF has a deep influence also on the mechanical properties of PET, as well as on PET recycling, where the chemical and morphological changes that lead to higher fragility and lower ductility of the polymer have been rationalized, taking into account the fact that a higher RAF is established upon recycling: the chain segments that constitute the MAF are more sensitive to the thermal degradation occurring upon PET recycling, which takes place via chain scissions and leads to a reduction in molar mass. Shorter amorphous chains can rearrange more easily into crystalline domains, thus increasing both the crystalline and rigid amorphous fractions [263].

**Figure 9:**
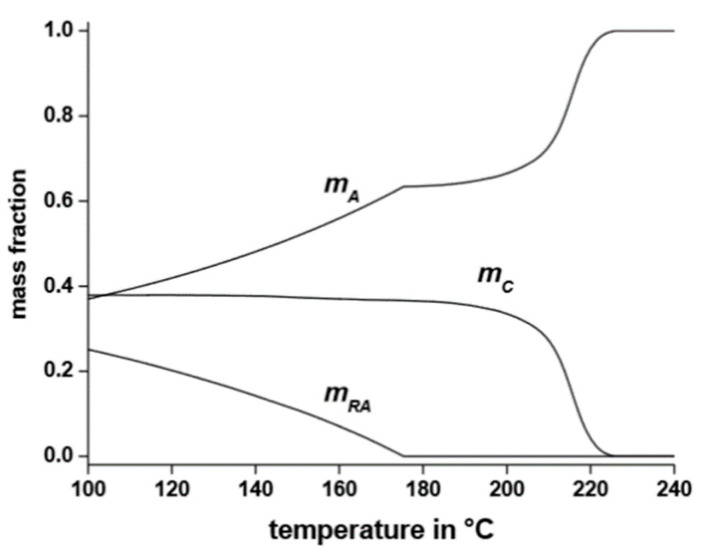
Mobile amorphous (*m*_A_), rigid amorphous (*m*_RA_), and crystalline (*m*_C_) mass fraction of PET upon cooling from the melt at 2 K/min. Reprinted from Ref. [265].

The formation of a rigid amorphous fraction is of huge importance in polymer crystallization, and various investigations of the mutual influence of the vitrification of the RAF and crystallization in PET have been conducted. The kinetics of RAF formation in PET was monitored during cooling from the melt, either upon a constant cooling rate, or upon quasi-isothermal step cooling [264,265,266]. The development of the three-phase structure of PET during continuous cooling at 2 K/min is illustrated in Figure 9. Apart from the cusps that derive from approximations in the analytical method, the non-isothermal cooling profile sheds light on the relationship between the vitrification of the RAF and crystal ordering: crystallization upon cooling from the melt at 2 K/min starts at 227 °C, with the major development of crystals, which takes place down to 170–180 °C, followed by an additional, slight increase in crystallinity with a further decrease in the temperature. On the other hand, the rigid amorphous structure starts to vitrify around 175 °C, i.e., during the final stages of non-isothermal crystallization, and completes vitrification after the end of crystallization, during the subsequent cooling to room temperature [264]. The vitrification of the RAF after the completion of primary crystallization is due to the temperature-dependent changes in chain mobility of the amorphous segments located in proximity of the crystal lamellae [264,265,266].

**Figure 10:**
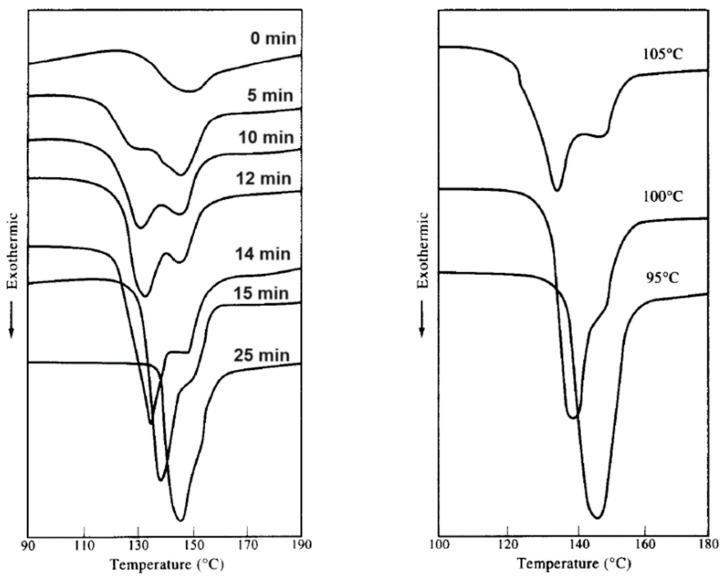
Cold crystallization exotherms of PET upon heating at 20 K/min after annealing at 105 °C for the indicated times (**left**) or after annealing at the indicated temperatures for 10 min (**right**). Adapted from Ref. [267], Copyright (1997), with permission from Elsevier.

The vitrification of the RAF was claimed to be the origin of a dual crystallization exotherm behavior in PET upon cold crystallization, as shown in Figure 10 [267,268]. A dual crystallization profile caused by the establishment of the RAF is not peculiar to PET, having been also reported for other polymers, like poly(butylene terephthalate) [269] or poly(3-hydroxybutyrate) [270]. The data shown in Figure 10 compare the cold crystallization exotherms of an initially amorphous PET, with PET partially crystallized at 105 °C for various times (left plot) or partially crystallized at 95–105 °C for 10 min and then heated at 20 K/min (right graph). A bimodal crystallization profile is visible in almost all the DSC plots of Figure 10, either as a shoulder or as a double-peaked exotherm. The initial growth of PET crystals is accompanied by the vitrification of the RAF, and the rigid amorphous chain portions coupled with the crystals hinder chain rearrangements at the amorphous/crystal growth front, which delays crystallization. The rise in temperature upon heating at 20 K/min leads to devitrification of the rigid amorphous segments at the crystal/amorphous interphase, whose increase in mobility facilitates chain ordering. This results in the high temperature exotherm shoulder in the DSC plot that reveals additional crystal formation.

**Figure 11:**
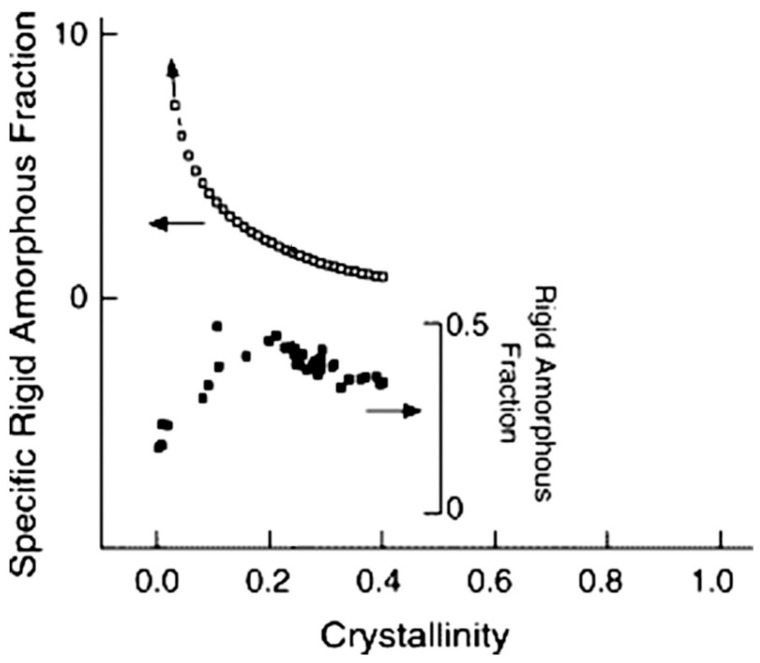
Rigid amorphous fraction of PET (filled squares, inset right axis), and specific rigid amorphous fraction (open squares, left axis) as function of crystallinity. Adapted from Ref. [271], Copyright (2005), with permission from Elsevier.

The amount of vitrified RAF in PET is determined by its thermal history and is linked to the crystal fraction, with a non-monotonous relationship. This is sketched in Figure 11, where the RAF is plotted as function of crystallinity for a PET that was cold-crystallized at 117 °C for various times and then annealed at higher temperatures. This thermal treatment led to a maximum detectable crystallinity *w*_C_ = 0.40 and a RAF that displays a maximum at *w*_C_ = 0.24 and then decreases at higher *w*_C_. The same plot also shows the trend in the specific RAF, that is the RAF normalized by crystallinity [271], which provides information on the average amount of rigid amorphous structure per unit of crystal. At the beginning of crystallization, the specific RAF displays its largest value and then continuously decreases with the progress of the phase transition. This trend is not peculiar to PET, having been noticed in a number of polymers both upon melt and cold-crystallization; the continuous decrease in the specific RAF with the progress of crystallization is due to crystal perfection and secondary crystallization, which decrease the strain transmitted to the amorphous phase, resulting in a decreased RAF/crystal ratio [59].

Figure 11 also highlights the link between RAF vitrification and secondary crystallization. The latter includes a variety of thermal processes that increase crystallinity after the completion of primary crystallization, i.e., all processes that lead to further crystal formation that are not associated with chain-folding lamellar growth [272,273,274,275]. Secondary crystallization occurs to a large extent in PET; hence, it plays a crucial role in determining the crystallinity and crystal morphology of PET [21,145,276,277,278,279,280,281,282,283,284,285,286].

Secondary crystallization mostly involves a reorganization/thickening of the crystal lamellae, as well the formation of tiny crystals within amorphous regions [55]. Various models have been hypothesized to describe the latter process, which mostly foresee the insertion of thin lamellae into the interlamellar region, or the insertion of thin lamellar stacks between stacks of lamellae developed upon primary crystallization [276]. In all cases, these processes take place in geometrically restricted areas, where polymer chains are under large constraints, and involve the areas close to the fold surface of the lamellae [277,278]. This implies a link between secondary crystallization and the vitrification of the rigid amorphous fraction probed in PET [59,265,266,279].

In isothermal melt crystallization of PET, primary and secondary crystallization overlap since the early stages of lamellae development [280]. The two processes have different time dependences, determined as an exponential increase, and an increase with the square root of time for primary and secondary crystallization processes, respectively. The varied time dependence arises from the critical growth nuclei: in the primary process, growth is nucleated on the edge surface, hence is limited by chain entanglements, whereas, in the secondary process, growth nucleates on the top or bottom surfaces and is determined by local reptation. The differences between the two surface free energy terms accounts for the large difference in the thicknesses of the nuclei and diffusion mechanisms by which the chain segments are laid on the growth surfaces and result in the growth of the primary process being linear with time, while secondary process depends on the square root of time [280,281].

The multiple processes overlapping during heating of PET lead to the very complex melting behavior of this polymer, as it displays multiple endotherms when analyzed by differential scanning calorimetry, which are not linked to crystal polymorphism. The variety of processes occurring in a narrow temperature range involves both the crystals and the coupled amorphous chain portions that constitute the RAF. The latter is established upon cooling/crystallization and devitrifies during heating. The exact *T*_g_ of the RAF is difficult to be directly identified, not only because it is largely affected by the thermal history that determines the coupling of the RAF with the lamellar structure [59], but mostly because RAF devitrification is associated with a continuous change in the coupled crystallized chain segments, which progressively anneal/perfect during heating. Most often, mobilization of the RAF takes place in a very wide temperature range, often overlapping with crystal melting [58]. This has raised a debate on a possible connection between crystal melting and RAF mobilization: some authors claim that the RAF devitrifies before the onset of melting [21], others say instead that PET crystals must melt before the RAF can mobilize [287].

However, the coupling between PET crystals and rigid amorphous portions contributes to the complexity of the PET melting profile and causes the appearance of double or triple endotherms upon DSC heating at conventional rates. For isothermally crystallized PET, a small endotherm appearing a few degrees above *T*_c_ upon subsequent heating, has been shown to be due to the latent heat associated with the melting of a small crystal fraction, plus the enthalpy recovery that accompanies the partial mobilization of the RAF [288]. Rearrangements of PET crystals at high temperatures involve the recrystallization/annealing/crystal perfection that goes along with partial melting. The latter can take place only when the amorphous chain portions coupled to the crystals have sufficient mobility, i.e., above the *T*_g_ of the RAF [289].

Similar to other polymers (e.g., poly(butene-1) [290,291], poly(lactic acid) [292,293], or poly[(R)-3-hydroxybutyrate] [270,294]), a limit temperature for the disappearance of the rigid amorphous fraction has been identified in PET, and is 215 °C [295,296]. This temperature has been associated with the transition between the double and triple melting behavior of PET after isothermal crystallization, discussed above, highlighting the link between the vitrification of the RAF, crystal formation and perfection, and crystal melting.

## 8. Conclusions

In this review, a comprehensive summary of the state-of-the-art knowledge of the crystallization behavior of PET is provided. It includes information about the kinetics of crystallization and the crystal structure and morphology developed according to the crystallization pathway. PET can be crystallized from the melt over a wide range of supercooling conditions or via quenching to its amorphous state, followed by thermal treatment above the glass transition temperature, to tailor crystal fraction and density.

The peculiarities of PET crystallization are presented and discussed. These mostly arise from the production of this polyester via polycondensation and from the effects of the catalyst residues and side products, mainly diethylene glycol, that are included as comonomers in the main chain, and from the formation of side branches. The latter are often purposely designed in PET production to favor processability, e.g., by reaction with chain extenders to produce PET foams, or arise from recycling and processing the post-consumer polymer. In fact, PET is an environmentally friendly polymer thanks to the economically feasible technologies used to recover/recycle post-consumer PET as an alternative to traditional disposal into landfills, as well as to the possibility of producing the polymer from bio-based monomers.

The wide application range of PET, either as virgin material or as recycled polymer, derives from its easily tunable crystallization kinetics and morphology, which in turn allow us to tailor its material properties. Therefore, a thorough understanding of the main factors that contribute to its varied crystallization behavior, as reviewed in this contribution, is essential for the proper design of PET processing.

## Data Availability

Data sharing is not applicable to this article.
